# Principled XAI analysis of the deep learning-based landslide susceptibility prediction model

**DOI:** 10.1038/s41598-026-52786-z

**Published:** 2026-06-14

**Authors:** Jongchan Oh, Jung-Hyun Lee, Hyuck-Jin Park, Daeung Yoon

**Affiliations:** 1 Department of Energy Resources Engineering, Chonnam National University, Gwangju, 61186 Republic of Korea; 2 Department of Geoinformation Engineering, Sejong University, Seoul, 05006 Republic of Korea

**Keywords:** Landslide susceptibility analysis, Machine learning, Deep learning, Convolutional neural networks, eXplainable Artificial Intelligence, Environmental sciences, Hydrology, Natural hazards, Solid Earth sciences

## Abstract

Research on applying machine learning (ML) and deep learning (DL) techniques to landslide susceptibility analysis is widespread, with increasingly accurate analyses through novel models. Predicting landslide susceptibility using ML models involves analyzing relationships between conditioning factors and landslide occurrences. Unlike traditional methods, ML models do not explicitly incorporate geotechnical or hydrological theories, raising concerns about result reliability despite high accuracy. This “black-box” limitation has prompted research applying eXplainable Artificial Intelligence (XAI) algorithms to interpret relationships between conditioning factors (digital elevation models (DEM), forest characteristics, soil properties, and geological features) and landslide susceptibility, thereby validating proposed ML models. In this paper, landslide susceptibility prediction models were developed using 20 conditioning factors and multiple architectures, including Support Vector Machine (SVM), Random Forest (RF), Multilayer Perceptron (MLP), and Convolutional Neural Networks (CNNs). Quantitative performances and XAI outcomes were compared. Specifically, the quantitative evaluation showed that the traditional point-based models (RF, SVM, and MLP) achieved Accuracies of 0.6931, 0.6621, and 0.7034, respectively, while the image-based CNNs achieved a higher Accuracy of 0.7586. Furthermore, regarding Recall—a critical metric for disaster management to minimize false negatives—the CNNs (0.8138) significantly outperformed the RF (0.6345), SVM (0.6276), and MLP (0.6828). These results underscore that capturing spatial context through image-wise inputs is far more effective for landslide susceptibility mapping than conventional pixel-level analysis. Because CNNs process input data differently, Gradient-weighted Class Activation Mapping (Grad-CAM) was applied alongside SHapley Additive exPlanations (SHAP) for CNNs, whereas only SHAP was applied to the other models. Results indicated specific patterns associated with certain conditioning factors in landslide susceptibility prediction. CNNs’ Grad-CAM heatmap effectively illustrated these patterns by treating data as images, improving interpretability and reliability of ML outputs.

## Introduction

Landslides are disasters that occur when the slopes of a specific area have unstable conditioning factors and encounter triggering factors such as rainfall or earthquakes^1,2^. Due to the massive damage they cause, extensive research has been conducted on landslide susceptibility analysis to predict and prevent such events^3–5^. Landslide susceptibility analysis is generally performed using a Geographical Information System (GIS) and can be divided into deterministic and statistical methods^6–8^. Deterministic methods involve performing susceptibility analysis using theoretical physical relationships, such as the factor of safety (FS) equation, with an infinite slope model^9,10^. These methods are also applied through the combination of physical slope instability and hydrological modeling, where the selection of parameters alters the purpose and results. Many studies have been conducted, such as Shallow Landslide STABility (SHALSTAB)^11,12^, Distributed physically based slope stability model (dSLAM)^13,14^, Transient Rainfall Infiltration and Grid-Based Regional Slope Stability (TRIGRS)^15^, Shallow Landslide Instability Prediction (SLIP)^16^. However, as the study area increases in size, the number of parameters to be calculated increases, which restricts deterministic methods mainly to local scale (≥ 1:5000), limiting their application to broad areas. The statistical method takes into account the uncertainty of data and estimates the correlation between conditioning factors and landslides rather than performing direct calculations, allowing for application across different scales^17–19^. Saha et al. (2005) applied landslide susceptibility zonation to the extensive Himalayas using the information value (InfoVAL) and weighted-based Landslide Nominal Risk Factor (LNRF)^20^. Additionally, methods such as fuzzy logic and the analytical hierarchy process (AHP)^21^, logistic regression^22–26^ have been applied.

ML techniques, which are fundamentally statistical in nature, have demonstrated high accuracy in landslide susceptibility analysis. Numerous studies have employed various algorithms, including decision trees^27^, artificial neural networks (ANNs) either standalone or integrated with fuzzy logic^21^, 28]–^31^, support vector machines (SVM)^5,32^, random forests (RF)^33^, neuro-fuzzy models, and ensemble methods^34–36^. Furthermore, boosting algorithms such as Light Gradient Boosting Machine (LightGBM) and eXtreme Gradient Boosting (XGBoost) represent widely adopted tools for landslide susceptibility assessment^37^. To improve generalization performance, several studies have recently applied transfer learning (TL) techniques in landslide susceptibility mapping^38–40^. For instance, Su et al. (2025) conducted multi-source domain adaptation using TL based on Maximum Mean Discrepancy (MMD) loss analysis^39^. Additionally, Wang (2025) performed 3D slope stability analysis via Scoops3D for non-landslide sampling and enhanced TL performance by quantifying the source-target similarity index through confidence ellipse-based regional similarity evaluations^40^. However, despite the high accuracy of methods using ML, questions regarding the reliability of the results of susceptibility analysis are often raised because theoretical relationships are not used in the predictions^41^. Additionally, because the ML is a black-box model, it suffers from poor explainability. To address this, eXplainable Artificial Intelligence (XAI) techniques are being applied^42–44^. In another recent advancement, Wang (2025) performed regional-scale spatial extrapolation using TL and subsequently utilized SHapley Additive exPlanations (SHAP) to provide a robust interpretability framework for the model’s predictions^45^. XAI is an algorithm proposed to provide explainability to ML models. When using ML models, XAI addresses uncertainty by making decisions trustworthy for users without offering any predetermined rules or formulaic regularization. By applying XAI, one can gain detailed information about how the trained model made predictions, how it predicts, and how it will predict in the future^46,47^. In this paper, XAI techniques were applied to trained ML models to increase the reliability of the landslide susceptibility predictions and reduce uncertainty.

Convolutional Neural Networks (CNNs) are also among the deep learning (DL) algorithms applied in landslide susceptibility analysis and have recently been evaluated as performing better than other ML and DL models^48–51^. Moreover, ML models trained in a point-wise manner are limited in their ability to capture the influence of surrounding spatial context during the training process. In contrast, Convolutional Neural Networks (CNNs), which are trained in an image-wise manner, can effectively account for such complex spatial dependencies. Furthermore, Wang et al. (2023) demonstrated the effectiveness of deep TL by utilizing image-wise inputs, highlighting the significance of capturing spatial context in deep learning-based susceptibility analysis^38^. However, despite the strong predictive performance of CNNs, research on the application of explainable AI (XAI) techniques to these models remains limited. Therefore, this paper aims to enhance the interpretability and reliability of CNNs by applying XAI algorithms, specifically SHAP and Grad-CAM. Additionally, the quantitative predictive performance of CNNs was compared with that of the models traditionally used, to evaluate the performance of the proposed CNNs model.

The primary contributions of this study are as follows:

1. We utilized image-wise CNNs to mitigate the limitations of point-wise inputs, which often fail to capture the spatial patterns required for accurate landslide susceptibility mapping.
2. We enhanced the interpretability and reliability of the DL model’s results by applying Grad-CAM and SHapley Additive exPlanation (SHAP) to the image-wise CNNs architecture.
3. We demonstrated the model’s ability to interpret spatial continuity through a rigorous spatial analysis of the trained model, proving its superiority over conventional point-based methods.

This paper is organized as follows: Sect.  2 provides a description of the study area and data, and Sect.  3, on methodology, details the ML models used, XAI algorithms, and evaluation metrics. Subsequently, the susceptibility mapping results, XAI analysis, and performance evaluation of the trained models are presented in Sect.  4. Finally, the conclusions and summary are discussed in Sect.  5.

### Study area and data

#### Study area

**Fig. 1:**
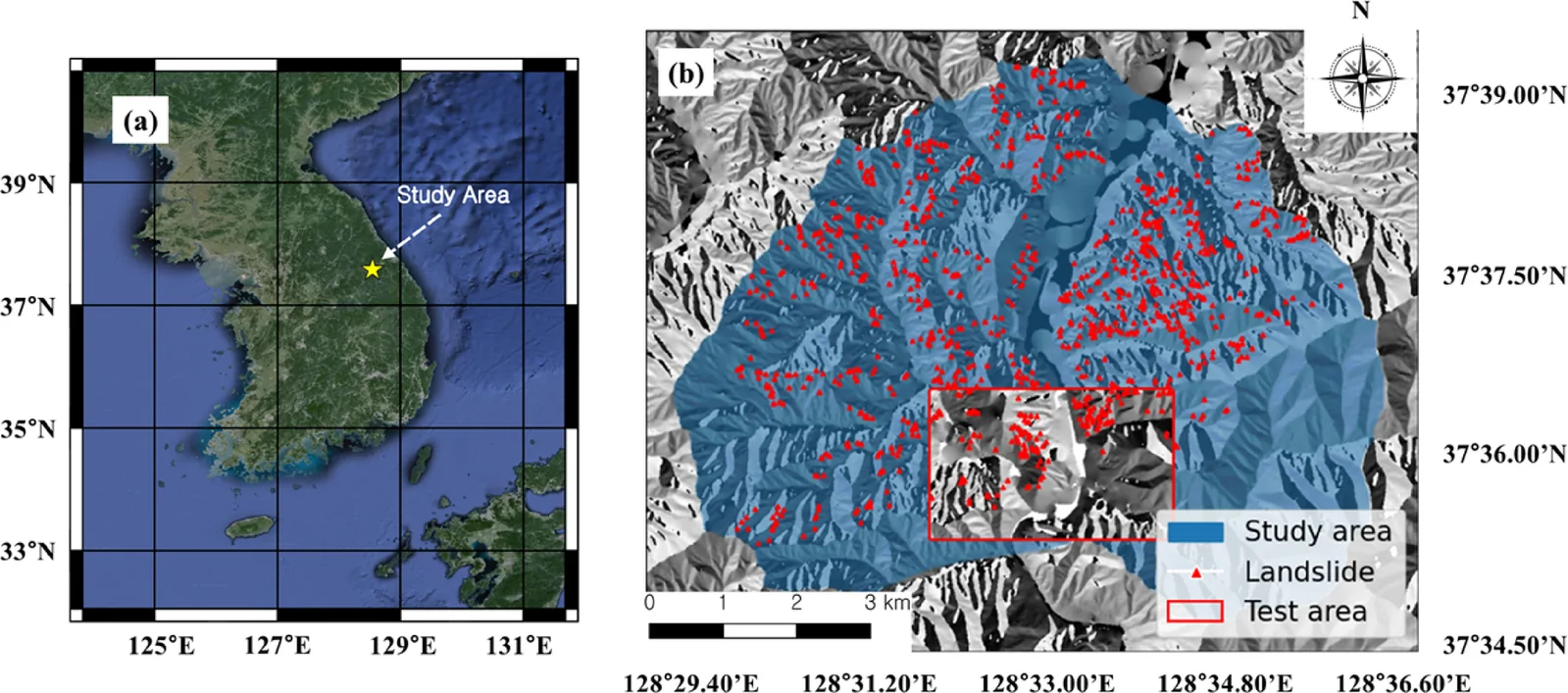
(**a**) Geographical location of the study area, Jinbu-myeon in Pyeongchang-gun, Gangwon-do, South Korea. (**b**) Shape of study area, distribution of landslide locations (totaling 1,035 red triangle points), and test region (red box). The maps were generated using QGIS (version 3.32.0, https://www.qgis.org/).

The study area, Jinbu-myeon in Pyeongchang-gun, Gangwon-do, South Korea (Fig. 1(a)), experienced multiple landslides in July 2006^52^. The total area is approximately 64–74 km2, with over 78% of the region consisting of mountainous terrain (Fig. 1(b)). We completely divided the test area from the study area to prevent data leakage and confirm the reliability of the model’s generalization performance. Since patch-wise inputs can overlap, data leakage–meaning the test datasets contain training (or validation) data–may occur.

**Table 1:** Summary of the original scale of the spatial data and data source.

Data Layer	Conditioning factors	Original Scale	Data source
Topographic Map (DEM)	Aspect, Slope, Elevation, Curvature (Planform, Profile, Standard), SCA, SPI, TWI	1:5,000	National Geographic Information Institute
Geological Map	Geology	1:50,000	Korea Institute of Geoscience and Mineral Resources
Forest Map	Forest age, Forest density, Forest diameter, Forest type	1:25,000	Korea Forest Service
Soil Map	Soil drainage, Soil series, Soil sub-texture, Soil texture, Soil thickness	1:25,000	National Institute of Agricultural Sciences
Land Use Map	Land use	1:5,000	Ministry of Environment

### Data acquisition

The landslide inventory was constructed using 0.5 m resolution satellite maps from Google Earth. Areas were classified as landslides if the extent of vegetation removal on the surface exceeded a certain area before and after the heavy rain in July 2006. The landslide inventory used in this study was established by the research team led by Park (2013), who are co-authors of this work^19^. To ensure highest data precision, landslide locations were identified through a rigorous visual interpretation of high-resolution aerial photographs (50-cm ground resolution) provided by the National Geographic Information Institute (NGII) and Samah Aerial Survey Co. Ltd. These images were captured using an UltraCam-X sensor on April 4, 2005 (pre-event), and May 27, 2008 (post-event). The reliability of this remote sensing-based inventory was further enhanced through systematic field-validation surveys conducted by the team to confirm the landslide occurrences. A total of 1,035 landslide locations were identified. Conditioning factors used in the study include nine factors derived from the DEM (Digital Elevation Model) provided from the NGII: elevation, planform curvature, profile curvature, standard curvature, specific catchment area (SCA), slope angle, slope aspect, stream power index (SPI), and topographic wetness index (TWI). Additionally, a geology factor provided by KIGAM (Korea Institute of Geoscience and Mineral Resources); four forest factors from the KFS (Korea Forest Service): forest type, forest density, timber age, and timber diameter; five soil factors provided by the National Institute of Agricultural Sciences (NIAS): soil drainage, soil series, soil sub-texture, soil texture; and land-use based on the land cover map provided by the MoE (Ministry of Environment) were used. The landslide conditioning factors were derived from spatial and geotechnical datasets originally compiled by Park et al. (2013) and subsequently utilized by Lee et al. (2020)^19,52^. To ensure transparency, the sources and original scales of the spatial data are summarized in Table 1. All input layers were digitized using QGIS (Quantum Geographic Information System) and standardized to a common grid resolution of 10 m × 10 m. This cell size was selected to match the 1:5,000 scale topographic base map while maintaining a balance between capturing critical terrain features and acknowledging the inherent resolution limits of the 1:25,000 and 1:50,000 thematic maps (i.e., avoiding pseudo-precision). A 10 m resolution DEM was constructed using a Triangulated Irregular Network (TIN) approach based on contour lines and elevation control points from the 1:5,000 topographic map.

Fig. 2Maps illustrating the 20 landslide conditioning factors employed in this study: (**a**) geology types, (**b**) slope aspect (directional orientation), (**c**) elevation, (**d**) slope angle, (e) planform curvature, (**f**) profile curvature, (**g**) standard curvature, (**h**) specific catchment area (SCA), (**i**) stream power index (SPI), (j) topographic wetness index (TWI), (**k**) forest age, (**l**) forest density, (**m**) forest diameter, (n) forest type classification, (**o**) soil drainage capability, (**p**) soil series, (**q**) soil sub-texture (20–100 cm depth), (**r**) soil texture (0–20 cm depth), (**s**) soil thickness, and (**t**) current land-use classifications. All factors were digitized at 10 m resolution. The maps were generated using QGIS (version 3.32.0, https://www.qgis.org/). Aspect, Slope, Elevation, Curvature, (Planform, Profile, Standard), SCA, SPI, TWI.

**Figure:**
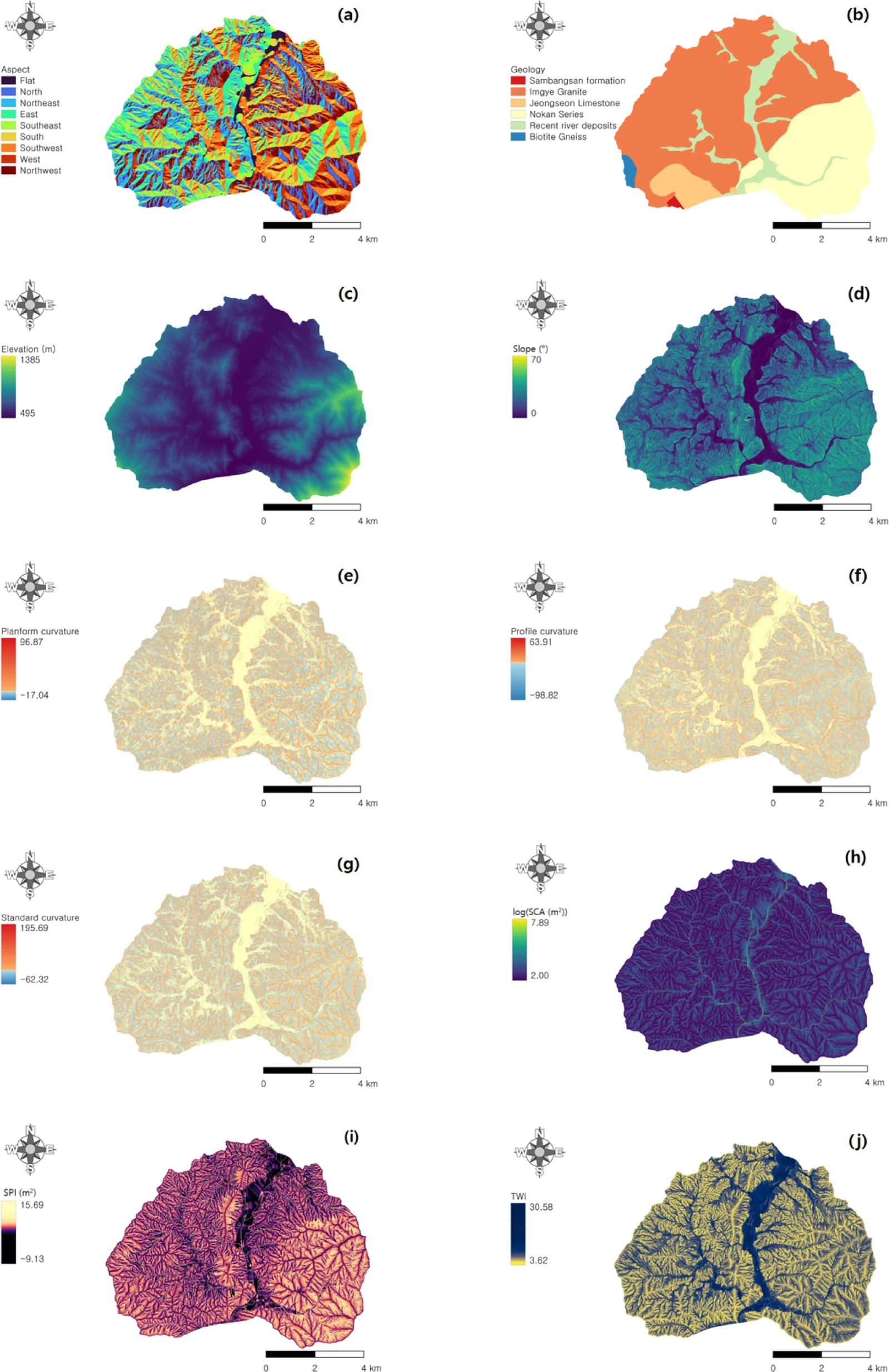

**Figure:**
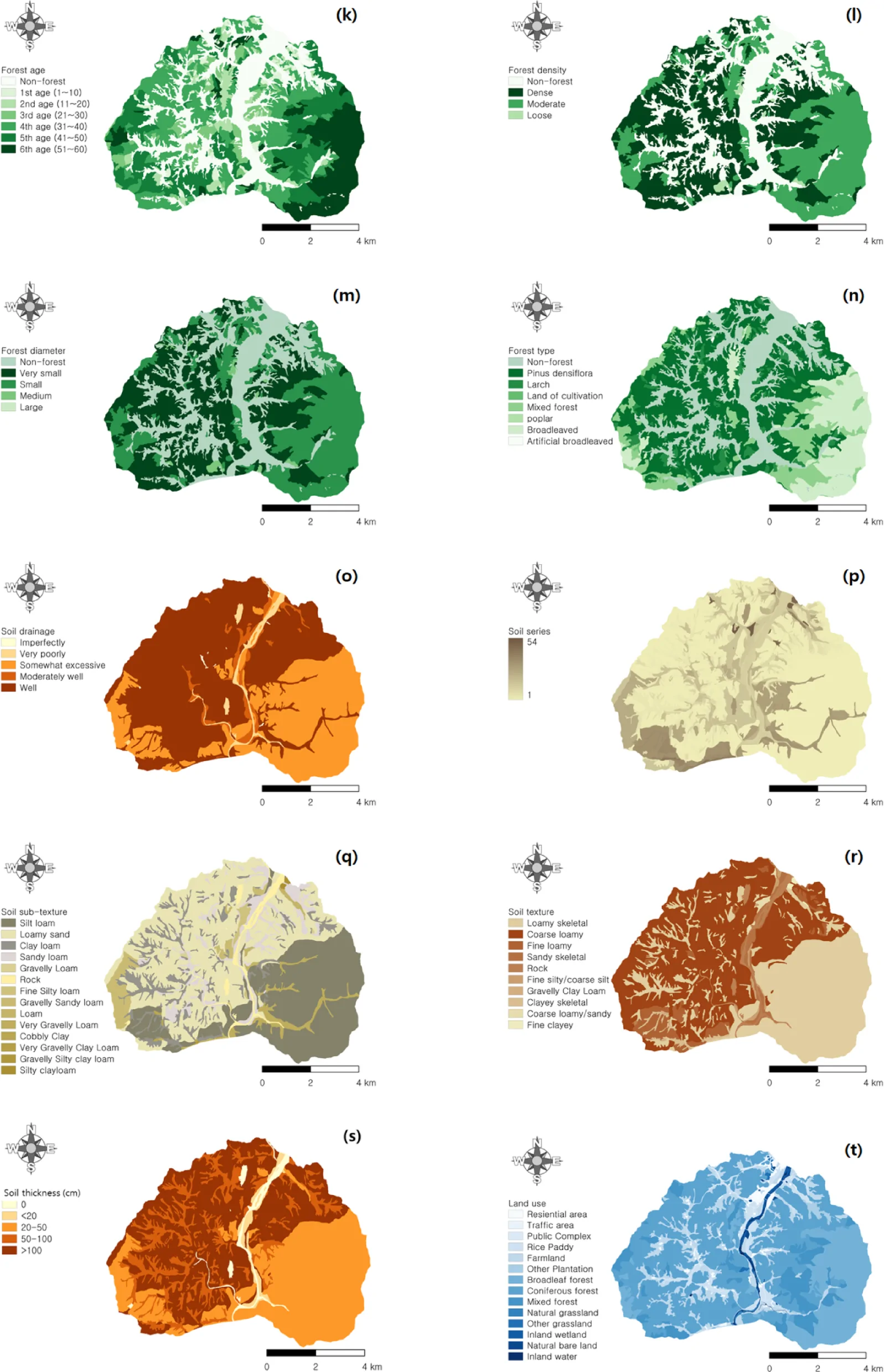


In this paper, we used these 20 characteristics to perform ML-based landslide susceptibility analysis. Geology represents the distribution of rocks and structures exposed at the surface, and certain rock types are inherently more susceptible to failure^53^. The characteristics of the factors derived from the DEM are as follows. Aspect refers to the orientation of a slope, and depending on this orientation, factors such as wind exposure and solar radiation can accelerate rock weathering, which may, in turn, increase the likelihood of landslide occurrence^54,55^. Slope and elevation refer to the angle of the slope and the altitude above sea level, respectively. Slope angle is directly related to slope stability and is often highly correlated with landslide occurrence within certain ranges. Elevation is also associated with slope stability, and although its influence may vary by region, it can affect landslide occurrence within specific elevation intervals^56^. Curvature provides information on how the slope bends; profile curvature affects the velocity of fluid flow parallel to the slope, while plan-form curvature influences the convergence and divergence of surface flow perpendicular to the slope that can affecting to landslide occurrence^57^. Standard curvature combines both profile and planform curvatures. Curvatures are derived using the following equations:

1 $$\:\begin{array}{c}\left\{\begin{array}{c}{C}_{Plan}=\frac{{f}_{xx}{{f}_{y}}^{2}-2{f}_{xy}{f}_{x}{f}_{y}+{f}_{yy}{{f}_{x}}^{2}}{({{f}_{x}}^{2}+{{f}_{y}}^{2}){(1+{{f}_{x}}^{2}+{{f}_{y}}^{2})}^{\frac{3}{2}}}\\\:{C}_{Prof}=\frac{{f}_{xx}{{f}_{x}}^{2}-2{f}_{xy}{f}_{x}{f}_{y}+{f}_{yy}{{f}_{y}}^{2}}{({{f}_{x}}^{2}+{{f}_{y}}^{2}){(1+{{f}_{x}}^{2}+{{f}_{y}}^{2})}^{\frac{3}{2}}}\\\:{C}_{std}=\frac{{\partial\:}^{2}z}{\partial\:{x}^{2}}+\frac{{\partial\:}^{2}z}{\partial\:{y}^{2}}\end{array}\right.\end{array}$$

where $\:{f}_{x}\:$ and $\:{f}_{y}$denote the first-order partial derivatives in each direction, $\:{f}_{xx}$, $\:{f}_{xy}$ and $\:{f}_{yy}$ represent the second-order partial derivatives.

SCA is defined as the area of the upslope contributing area per unit contour length and is used to describe complex terrain through the flow of fluid on slopes^58^. TWI is derived from the amount of water provided by the upslope area and local drainage, capturing the topographic influence on hydrological processes^59^. It can be calculated using the following equation:

2 $$\:\begin{array}{c}TWI=ln\left(\frac{{A}_{s}}{tan\beta\:}\right)\end{array}$$

where $\:{A}_{s}$ represents the SCA and $\:tan\beta\:$ denotes the local slope gradient in radians.

3 $$\:\begin{array}{c}SPI={A}_{s}\times\:tan\beta\:\end{array}$$

SPI is calculated using the catchment area and slope gradient, measuring the erosive power of water flow^60^. It is derived from the SCA and slope values through the following equation:

Forest type is broadly categorized into ten types, including coniferous, broadleaved, and non-forest areas. Forest age, diameter, and density are classified by the age of the forest in decades, the diameter of trees measured at breast height, and the proportion of the area covered by trees taller than 8 m, respectively. The type and extent of forest cover within a region are associated with the occurrence of landslides^61,62^. Soil drainage indicates how well the soil can absorb water during rainfall. Soil texture refers to the soil properties at a depth of 0–20 cm, and sub-texture refers to the soil properties at a depth of 20–100 cm^63^. Soil series classify soil based on similar characteristics to soils from other regions, and thickness refers to the depth to bedrock or the point where geotechnical and hydrological properties change^64^. Soil drainage refers to the extent to which soil retains water, which is influenced by factors such as soil series, sub-texture, texture, and soil thickness. The degree of water retention within the soil can increase or decrease the probability of landslide occurrence^65^. Finally, land use describes how an area is actually utilized, and changes in land use and the resulting alterations in land cover can potentially affect to landslide susceptibility^66^.

## Methodology

### Workflow

The workflow of the study is as follows. First, to evaluate the performance of the trained model, approximately 15% of the entire study area was randomly selected and allocated as the test region (Fig. 1(b)). This region contains 145 landslide points, and non-landslide points were generated through random sampling within the same area. Furthermore, the data from this test region were excluded from both the training and validation datasets. Next, we performed simple random sampling of the non-landslide data and image patching for the study area, and trained the ML models using the preprocessed dataset. Using the trained models, we conducted landslide susceptibility mapping and performance evaluation. Finally, we analyzed the trained models using XAI algorithms.

### Data preparation and sampling Strategy

The input data were processed into small patches to facilitate the training of the deep learning models. We employed a sliding window technique with an overlapping strategy to generate 12 × 12 pixel patches with a stride of 1, representing a ground area of 120 m×120 m. his patch size was determined empirically by testing multiple candidate sizes (e.g., 8 × 8, 9 × 9 and 10 × 10 pixels) and selecting the 12 × 12 configuration, which yield the most stable predictive performance. Through this process, a total of 644,365 patches were generated across the study area. To enhance the model’s ability to predict landslide susceptibility with high spatial precision, the label of each patch was determined based specifically on its central 2 × 2 grid cells. any of these central four cells contained a landslide point, the patch was labeled as 1 (landslide); otherwise, it was labeled as 0 (non-landslide), even if landslide points existed in the surrounding area of the patch. Furthermore, as a quality control measure, any patch where the central 2 × 2 grid cells contained NaN values or missing metadata was excluded from the analysis. This approach ensured that the model was trained on high-quality, representative samples, focusing on the precise locations of landslide occurrences.

To ensure transparency and reproducibility, we clarify the data preparation process for the CNNs and traditional machine learning models (RF, SVM, and MLP). First, to prevent data leakage, the study area was geographically partitioned into independent training and testing regions before sample extraction. Class imbalance was addressed by maintaining a 1:1 ratio between landslide and non-landslide samples through random under-sampling of the non-landslide class in both regions.

The total number of training samples was optimized according to each model’s architecture. For the image-based CNN, we implemented a spatial augmentation strategy using a sliding window with a stride of 1. Because the patch label is determined by the central 2 × 2 grid, a single landslide location can generate four distinct but spatially correlated patches. This approach resulted in 6,348 training samples for the CNN, enhancing its ability to recognize local spatial patterns without the physical inconsistencies often caused by traditional geometric augmentation (e.g., rotation). For the tabular-based models, which lack spatial context, we utilized only the primary landslide points corresponding to the exact centers of the training patches, resulting in 1,780 samples. This ensures that the point-wise data is a precise subset of the patch-wise connectivity.

Crucially, the evaluation of all models was conducted on a strictly identical test dataset to ensure an objective performance comparison. A specific area was reserved for testing, where 145 landslide and 145 non-landslide locations (total *n* = 290) were sampled. For the CNNs model, although it processes 12 × 12 patches, the test label was assigned based solely on the single central pixel (located at coordinate (5,5) within the patch), matching the point-wise samples used in the RF, SVM, and MLP models. This methodological rigor ensures that any disparity in predictive accuracy is purely a function of the models’ architectural capabilities in extracting topographic information rather than variations in the test data. Finally, to ensure numerical stability and consistency across all environmental variables, the input data were normalized using Min-Max scaling to rescale all values to a fixed range between 0 and 1.

### Machine learning models

In this paper, traditional ML techniques: Support Vector Machine (SVM) and Random Forest (RF) were used as classifiers to distinguish between landslide and non-landslide occurrences^67–69^. SVM is a model that solves classification problems by constructing linear or nonlinear decision boundaries based on the characteristics of the data, making decisions according to the following equation:

4 $$\:\:\begin{array}{*{20}c} {\left\{ {\begin{array}{*{20}c} {\vec{w} \cdot \overrightarrow {{x_{ + } }} + b \le \:\delta \:} \\ {\:\vec{w} \cdot \:\vec{x} + b = 0} \\ {\:\vec{w} \cdot \:\overrightarrow {{x_{ - } }} + b \ge \: - \delta \:} \\ \end{array} } \right.} \\ \end{array}$$

where $\:\overrightarrow{w}$ and * b* are the weight and bias of the model, respectively, and $\:\overrightarrow{{x}_{+}}$ and $\:\overrightarrow{{x}_{-}}$ represent samples located above and below the decision boundary. $\:\delta\:$ denotes the margin that defines the distance between these vectors. In the case of a hard margin, the two classes are perfectly separable, whereas in the case of a soft margin, some outliers are tolerated. In this paper, the Support Vector Machine (SVM) model utilized a radial basis function (RBF) kernel, which is effective for handling nonlinear classification problems. To ensure optimal performance, the regularization parameter *C* was set to 1000, and the kernel coefficient $\:\gamma\:$ was assigned a value of 0.001.

The Random Forest (RF) model is an ensemble-based method that constructs multiple decision trees by repeatedly sub-sampling the training data. Each tree is trained independently, and final predictions are made through averaging or majority voting of individual tree outputs. This approach enables robust classification performance by reducing variance. For the RF configuration in this study, the number of trees ‘*n_estimators’* was set to 30 with a maximum depth ‘*max_depth*’ of 15; notably, all available features were considered at each split ‘*max_features: None*’ to maximize the information gain from the high-dimensional conditioning factors.

Additionally, Multilayer Perceptron (MLP) and Convolutional Neural Networks (CNNs) were implemented. The MLP consists of one or more fully connected hidden layers and does not require manual feature engineering or prior statistical assumptions, allowing it to approximate highly nonlinear functions^70^. Regarding the MLP architecture, it was configured with two hidden layers, each containing 100 neurons. The Rectified Linear Unit (ReLU) was employed as the activation function, and the Adam optimizer was used for efficient weight updates. To mitigate the risk of overfitting, an L2 regularization parameter $\:\alpha\:$ of 0.0001 was applied. The MLP was trained with a batch size of 64 for up to 500 iterations to ensure stable convergence of the loss function. These specific hyperparameter sets were selected based on their superior predictive stability and accuracy during the empirical validation and grid search phase.

**Fig. 3:**
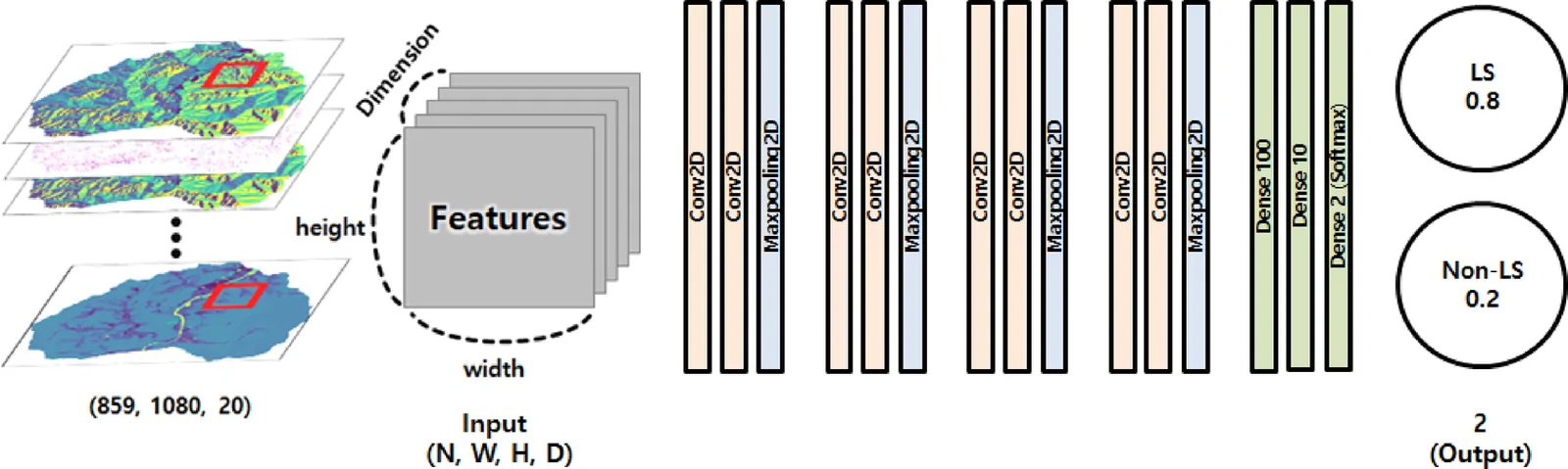
Detailed architecture of the Convolutional Neural Network (CNNs) used in this paper. The network consists of convolutional layers with ReLU activation to introduce non-linearity, followed by pooling layers and a fully connected layer with a softmax activation for binary classification. The input dimensions of the CNNs are (N: number of data samples, W: width, H: height, D: number of conditioning factors), and the output is a vector of size 2, representing landslide and non-landslide classes.

CNNs are networks specialized for image processing, consisting of convolution layers, pooling layers, and fully connected layers. By using kernels in the convolution layers, CNNs extract features from input images and learn complex patterns in the data^71–73^. In this paper, the input dimensions of the CNNs model are (N, 12, 12, 20), where N represents the number of data samples, W and H = 12 indicate the width and height of each input patch, and D = 20 corresponds to the number of conditioning factors. The model’s output is a vector of size 2, corresponding to the predicted probabilities of landslide occurrence and non-landslide, respectively (Fig. 3). We used the ReLU activation function between the convolution layers to maintain and enhance the non-linearity of the trained network^74^. A Softmax activation function was employed for the final layer. Although Softmax is mathematically equivalent to Sigmoid for binary classification, this two-neuron output structure was selected to facilitate the generation of class-specific Grad-CAM heatmaps. This approach allows for a more intuitive interpretation of the model’s focus on both ‘landslide’ and ‘non-landslide’ features. Additionally, categorical cross-entropy, a commonly used cost function for classification problems, was employed^75^.

### Permutation feature importance

Permutation feature importance is a technique used to analyze the contribution of each feature when a model is trained using multiple features^76^. The procedure begins by computing the model’s accuracy using the original dataset. Then, the values of a single feature are shuffled (permuted), and the model makes predictions using this modified dataset. The decrease in accuracy (calculated as the difference between the accuracy on the original data and that on the permuted data) reflects the importance of the shuffled feature. This process is repeated for all features, and the final feature importance rankings are determined based on the magnitude of the performance degradation caused by each permutation.

### SHapley additive exPlanations

SHAP is one of the XAI (eXplainable Artificial Intelligence) algorithms proposed to interpret machine learning techniques considered as black-box models. It analyzes which features of the input influence more to the output generated by the trained model^43,77^. The SHAP values are calculated by the following equation:

5 $$\:\begin{array}{*{20}c} {\phi \:_{i} \left( {\upsilon \:} \right) = \sum {\:_{{S \in \:N_{{left}} i}} } \frac{{\left| S \right|!\left( {n - \left| S \right| - 1} \right)!}}{{n!}}\left( {\nu \:\left( {S \cup \:i} \right) - \nu \:\left( S \right)} \right)} \\ \end{array}$$

where $\:{\phi\:}_{i}$ represents the SHAP value of the *i* th feature, *N* is the set of all features, *S* is a subset of *N* excluding *i* th feature, *n* is the number of features in the set *N*, and *v(S)* denotes the contribution of the feature within subset *S*. The SHAP value is calculated by systematically excluding each feature to determine how the value of that feature impacts the model’s output. Additionally, by averaging the absolute values of the SHAP values, the general contribution of each feature can be quantified. SHAP can also be applied to CNNs. In this case, the gradients of the trained CNNs model are calculated, and the gradient-based contribution for each input feature is quantified using Eq. (5) to estimate the SHAP values.

### Gradient-weighted class activation mapping

Grad-CAM is a generalized technique proposed by Selvaraju et al. (2020) to enhance the applicability of Class Activation Mapping (CAM), which was originally suggested by Zhou et al. (2016) for providing visual explanations of CNNs. When the class of an image with width u and height v is c, CAM can be calculated using the following Eqs.^78,79^:

6 $$\:\begin{array}{*{20}c} {\alpha \:_{k}^{c} = \frac{1}{Z}\sum {\:_{i} } \sum {\:_{j} } \frac{{\partial \:y^{c} }}{{\partial \:A_{{ij}}^{k} }}} \\ \end{array}$$

where $\:{y}^{c}$ represents the gradient when predicting class *c*, and $\:{A}^{k}$ refers to the corresponding convolution layer’s feature maps, which are obtained through backpropagation. $\:{\alpha\:}_{k}^{c}$ signifies the partial linearization weight during the prediction process and captures the importance of the feature map $\:{A}^{k}$ when predicting the target class *c*. These importance values are then subjected to global average pooling, as represented by the sigma in the equation.

Applying ReLU yields the final class-discriminative localization map known as Grad-CAM.

7 $$\:\:\begin{array}{*{20}c} {L_{{Grad - CAM}}^{c} = ReLU\left( {\sum {\:_{k} } \alpha \:_{k}^{c} A^{k} } \right)} \\ \end{array}$$

where, by applying ReLU to the linear combination term $\:{\alpha\:}_{k}^{c}{A}^{k}$, we focused on the parts that positively influence the specific class. In this paper, to identify which changes in conditioning factors affect landslides, we examined the pattern similarity between the Grad-CAM heatmap and the input conditioning factors.

### Evaluation metrics

To quantitatively evaluate the performance of the trained model, the AUC-ROC score was used as the accuracy metric. The AUC-ROC curve is plotted with specificity (False Positive Rate, FPR) on the x-axis and sensitivity (True Positive Rate, TPR) on the y-axis, reflecting the performance of the trained model^80^.

8 $$\:\begin{array}{*{20}c} {TPR = \frac{{TP}}{{TP + FP}}} \\ \end{array}$$

9 $$FPR = 1 - \frac{{TN}}{{TN + FP}}$$

The area under this curve is called the AUC score, and the closer the AUC score is to 1, the better the model’s performance in classifying the classes^81^. Additionally, Cohen’s Kappa was used as a metric to evaluate reliability of trained model^82,83^. Cohen’s Kappa measures the agreement between two observers and can be expressed using the following equation:

10 $$\:\begin{array}{*{20}c} {Accuracy = \frac{{TP + TN}}{{TP + TN + FP + FN}}} \\ \end{array}$$

Cohen’s Kappa was used as a metric to evaluate reliability of trained model^82,83^. Cohen’s Kappa measures the agreement between two observers and can be expressed using the following equation:

11 $${K = \frac{{Pr\left( a \right) - Pr\left( e \right)}}{{1 - Pr\left( e \right)}}}$$

where *K* is the Cohen’s kappa index, *Pr(a)* is the probability that both observers make the same judgment in the overall assessment, while *Pr(e)* is the probability that both observers make the same judgment by a chance. This indicates the likelihood of two observations being the same for a given input and is used as a quantitative metric for evaluation the reliability of model predictions in binary classification problems^83^.

In disaster contexts such as landslides, detecting actual occurrence events is more critical than identifying non-occurrence cases. Therefore, it is essential to ensure that the model does not miss potential hazard events. To specifically evaluate this capability, we adopted Recall (also known as Sensitivity), which focuses on minimizing False Negatives, as a key performance metric calculated as follows^84^:

12 $$\:\begin{array}{*{20}c} {\:\begin{array}{*{20}c} {Recall = \frac{{TP}}{{TP + FN}}} \\ \end{array} } \\ \end{array}$$

where TP (True Positive) represents the cases correctly predicted as landslides by the trained model, and FN (False Negative) denotes the cases where actual landslide events were incorrectly predicted as non-landslides. This metric measures the model’s effectiveness in identifying as many actual positive instances as possible without omission^84^.

Finally, the F1-score is a metric that combines precision and recall. It provides an indirect assessment of how well the trained model performs with imbalanced data and is calculated as follows^85^:

13 $$\:\begin{array}{*{20}c} {F1\:score = 2 \times \:\frac{{Precision \times \:Recall}}{{Precision + Recall}}} \\ \end{array}$$

For all four metrics, a score of 1 indicates the highest performance.

## Results

### Results of the traditional models

To evaluate the landslide susceptibility mapping performance, we trained and tested four models—SVM, RF, MLP, and CNN—using the optimized hyperparameter configurations detailed in the Methodology section. By applying the optimized configurations (e.g., the RBF kernel with *C* = 1,000 for SVM, 30 trees with a maximum depth of 15 for RF, and the two‑layer (100, 100) MLP architecture) consistently across all experiments, we ensured a fair and reproducible comparison. A comprehensive performance assessment of each model, including accuracy, AUC, kappa score, recall, and F‑score is presented in the Performance Evaluation subsection that follows.

**Fig. 4:**
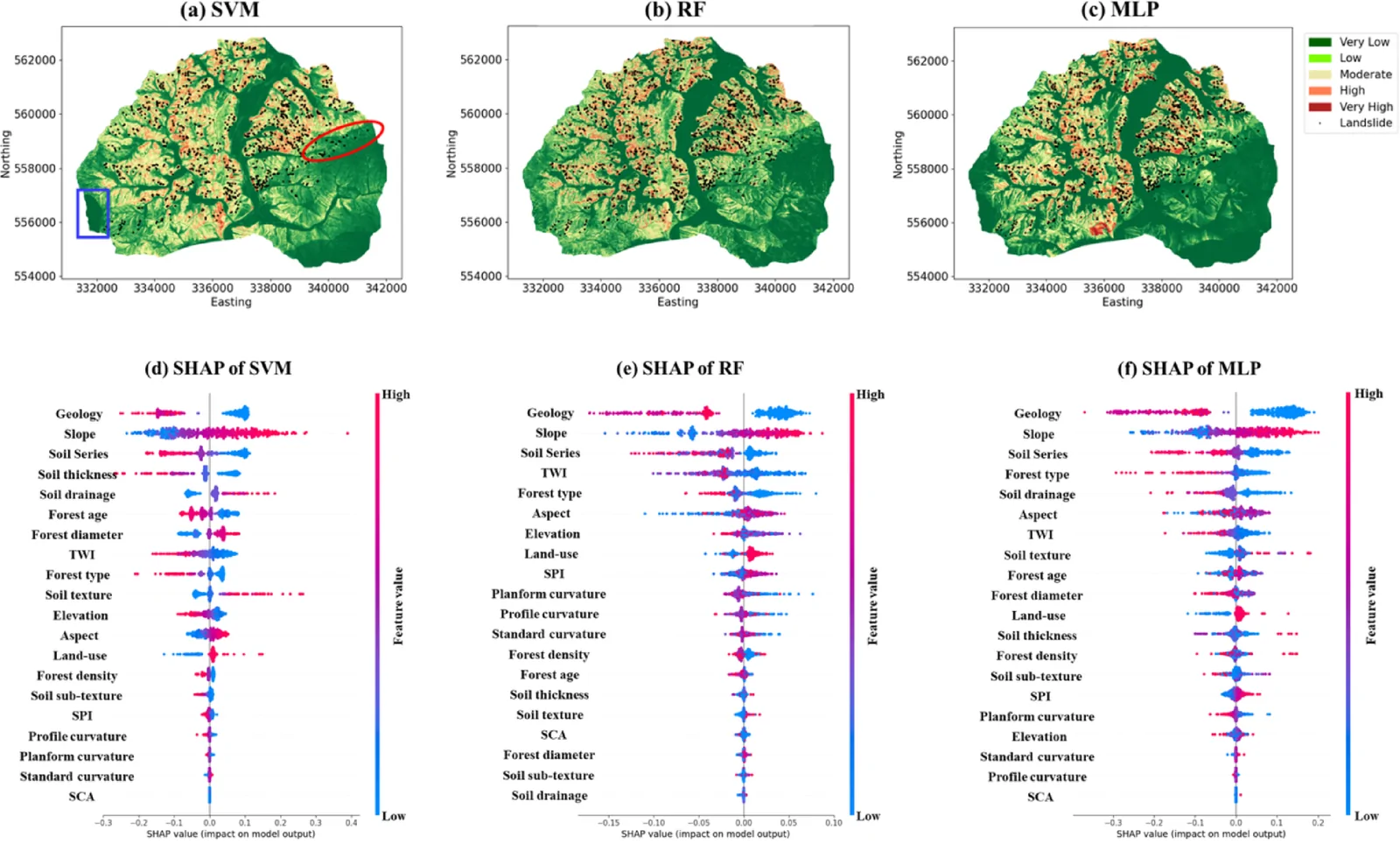
Landslide susceptibility maps generated by machine learning models and SHAP interpretation plots. Maps (**a–c**) illustrate the spatial distribution of susceptibility predicted by (**a**) Support Vector Machine (SVM), (**b**) Random Forest (RF), and (**c**) Multilayer Perceptron (MLP). Plots (**d–f**) present the corresponding SHAP summary plots for SVM, RF, and MLP, respectively. In the SHAP plots, red and blue dots represent higher and lower feature values, respectively. Geology, slope, and soil series were identified as the most influential factors among the conditioning factors. The maps were generated using Python 3 with the Matplotlib library (https://matplotlib.org/).

Using the trained SVM, RF and MLP models, susceptibility maps were generated for the study area (Fig. 4(a)-(c)). To ensure a systematic interpretation and spatial visualization of the results, the continuous susceptibility probability indices (ranging from 0.0 to 1.0) were categorized into five discrete levels. We adopted the Equal Interval classification scheme, defining the classes with a consistent increment of 0.2: Very Low (0.0–0.2), Low (0.2–0.4), Moderate (0.4–0.6), High (0.6–0.8), and Very High (0.8–1.0)^86^.

The choice of the Equal Interval method is justified by its ability to represent the models’ predicted probabilities on a consistent linear scale. Since this study involves a comparative analysis of multiple algorithms, maintaining a uniform threshold across all models is essential for an objective evaluation of the spatial extent and intensity of high-risk areas. This standardized categorization ensures that the resulting susceptibility maps reflect the intrinsic predictive characteristics of each model without being skewed by the underlying data distribution within the classes.

The SVM model appropriately predicted high susceptibility in the vicinity of some landslide points, as shown in Fig. 4(a). However, it underestimated susceptibility in certain landslide locations within the eastern region (highlighted by the red ellipse in Fig. 4(a)), assigning low probability values. Additionally, the model exhibited limited spatial continuity along the southwestern edge of the study area (indicated by the blue box in Fig. 4(a)). The RF model demonstrated somewhat improved continuity in edge areas where the SVM model underperformed. Nevertheless, similar to the SVM, it tended to predict low to moderate susceptibility around landslide points in the eastern area. Additionally, the overall susceptibility map produced by the RF model appeared scattered, resulting in reduced spatial coherence, as shown in Fig. 4(b). The predicted susceptibility map from the MLP model exhibited a pattern similar to that of the SVM model, showing limited spatial continuity along the edges of the southwestern and eastern regions. However, compared to traditional models, it predicted higher susceptibility values around landslide points and demonstrated slightly improved spatial coherence (Fig. 4(c)). Additionally, when evaluated on the test dataset, the SVM model achieved an accuracy of 0.6621, while the RF model yielded a slightly higher performance with an accuracy of 0.6931. The MLP model demonstrated the highest accuracy among the traditional models, reaching 0.7034.

**Fig. 5:**
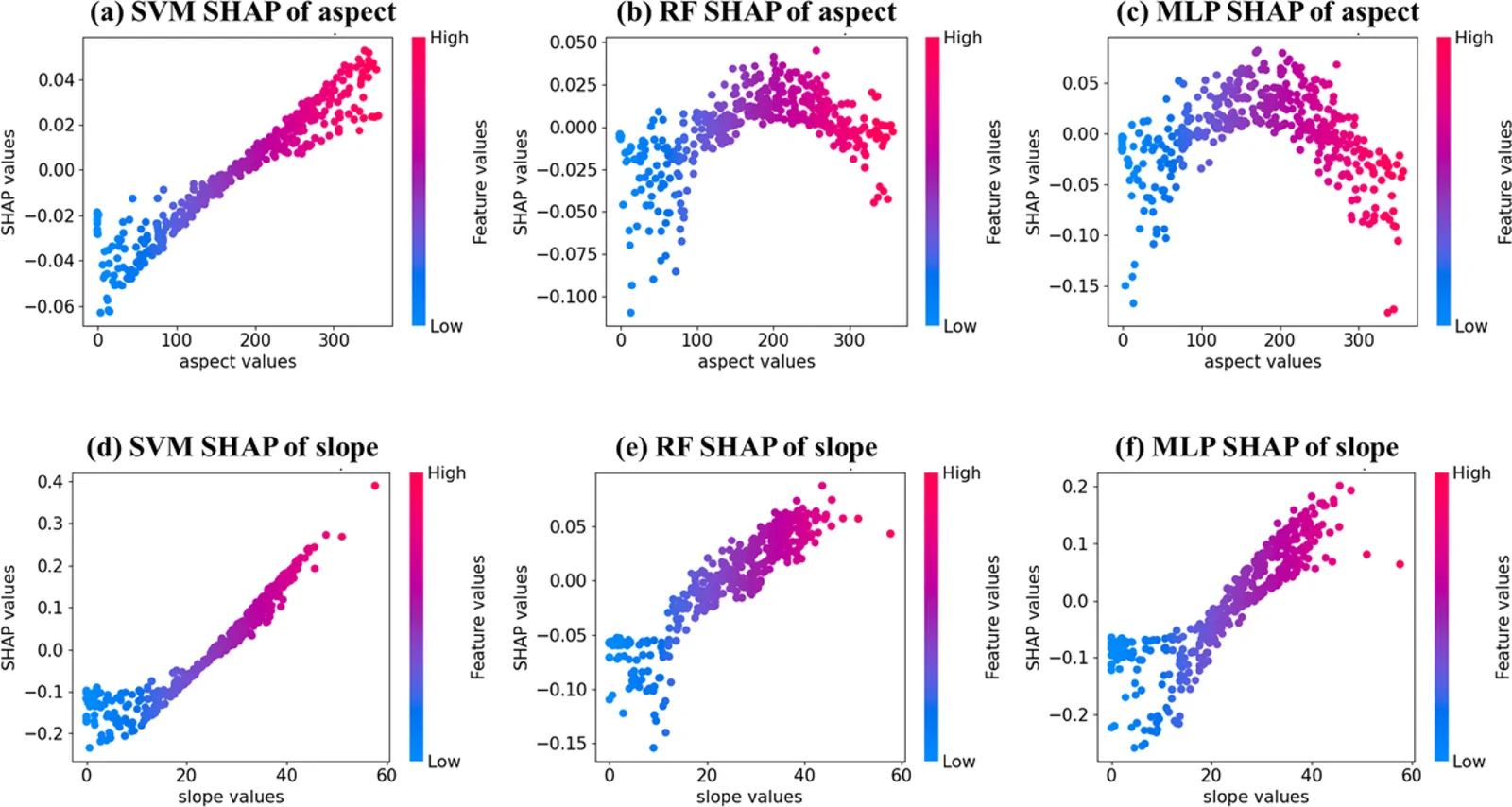
Scatter plots illustrating the relationships between SHAP values and two continuous conditioning factors (aspect and slope) for each traditional model: (**a**), (**d**) SVM; (**b**), (**e**) RF; and (**c**), (**f**) MLP. The plots reveal how variations in these factors influence the models’ landslide susceptibility predictions. Aspect values are interpreted in degrees, where 0° and 360° indicate north-facing slopes, 90° east-facing, 180° south-facing, and 270° west-facing.

The traditional machine learning models were analyzed using SHAP, as shown in Fig. 4(d)-(f). In the SHAP summary plots, the vertical axis (from top to bottom) represents the features ranked by their impact on the model’s predictions. The horizontal axis indicates the direction and magnitude of each feature’s contribution, with values on the left contributing toward class 0 (non-landslide) and values on the right contributing toward class 1 (landslide). Blue dots correspond to lower feature values, while red dots represent higher values. The SHAP analysis revealed that the top three most important features were consistent across all models, ranked in the order of geology, slope, and soil series (Fig. 4(d)-(f)). Based on these findings, it can be concluded that geology, slope, and soil series are among the most influential conditioning factors in landslide susceptibility prediction. Although the soil series factor ranked among the third most important factor in the SHAP results, it includes approximately 54 distinct soil series within the study area, making detailed interpretation difficult. Furthermore, since there are 405 soil series across South Korea, and each region contains a different subset of these, generalizing the results of this factor is challenging. In both the RF and MLP models, the forest type factor also showed high SHAP values. Specifically, higher SHAP values were generally associated with coniferous forests, while lower values were observed for broadleaf forests. This trend is closely related to the rooting characteristics of these vegetation types: coniferous trees tend to develop shallow, horizontally spreading roots, making them more vulnerable to external pressures such as strong winds or heavy rainfall. In contrast, broadleaf trees develop deeper, vertically oriented root systems that provide stronger resistance against such forces. For correlation analysis between model predictions and conditioning factors, continuous variables were deemed more suitable. While soil drainage ranked high in importance for both the SVM and MLP models, the direction of its contribution was inconsistent between them and showed the lowest SHAP values in the RF model. Therefore, it was excluded from the correlation analysis. The aspect factor, on the other hand, ranked sixth in both the MLP and RF models, and although it had relatively lower importance in the SVM model, a consistent pattern was still observed. As a result, aspect was selected along with slope for further correlation analysis (Fig. 5).

The correlation graphs in Fig. 5 illustrate how SHAP values vary depending on the magnitude of each conditioning factor. In these plots, the color and x-axis represent the factor values, while the y-axis indicates the corresponding SHAP values. For the SVM model, both the aspect and slope factors exhibit a linear increase in SHAP values as the factor values increase (Fig. 5(a), (b)). In contrast, for the RF and MLP models, the relationship between aspect and SHAP values follows a parabolic pattern: SHAP values are lowest around 0° and 360° (north-facing), moderate at 90° and 270° (east- and west-facing), and highest at 180° (south-facing), as shown in Fig. 5(c) and (e). For the slope factor, SHAP values increase linearly with slope angle in all three models, consistent with the trend observed in the SVM model (Fig. 5(b), (d), and (f)).

Since the geology factor is categorical, linear correlations could not be analyzed. Instead, its influence was examined by comparing the overall distribution of geological classes with the distribution of landslide occurrences. The geology factor consists of the following lithological units: Cs (Sambangsan Formation: 1), Gi (Imgye Granite: 2), Oj (Jeongseon Limestone: 3), TRn2 and TRn3 (Nokam Series: 4), Qr (Recent River Deposits: 5), and PCEbgn (Biotite Gneiss: 6). It was observed that the geological units Gi and TRn2 occupy a large proportion of the overall geology factor. Among the landslide occurrence data, only three units (Gi, Oj and TRn2) are represented. SHAP analysis results indicate that the majority of blue dots, which correspond to the unit Gi, are associated with high SHAP values, implying a strong positive contribution to landslide susceptibility. As a result, the model tends to predict lower susceptibility for data points whose geology is not classified as Gi. This distinct disparity in geological distribution between landslide and non-landslide areas highlights the significant influence of the geology factor, confirming its high importance in landslide susceptibility analysis (Fig. 4).

## Results of the CNNs model

**Fig. 6:**
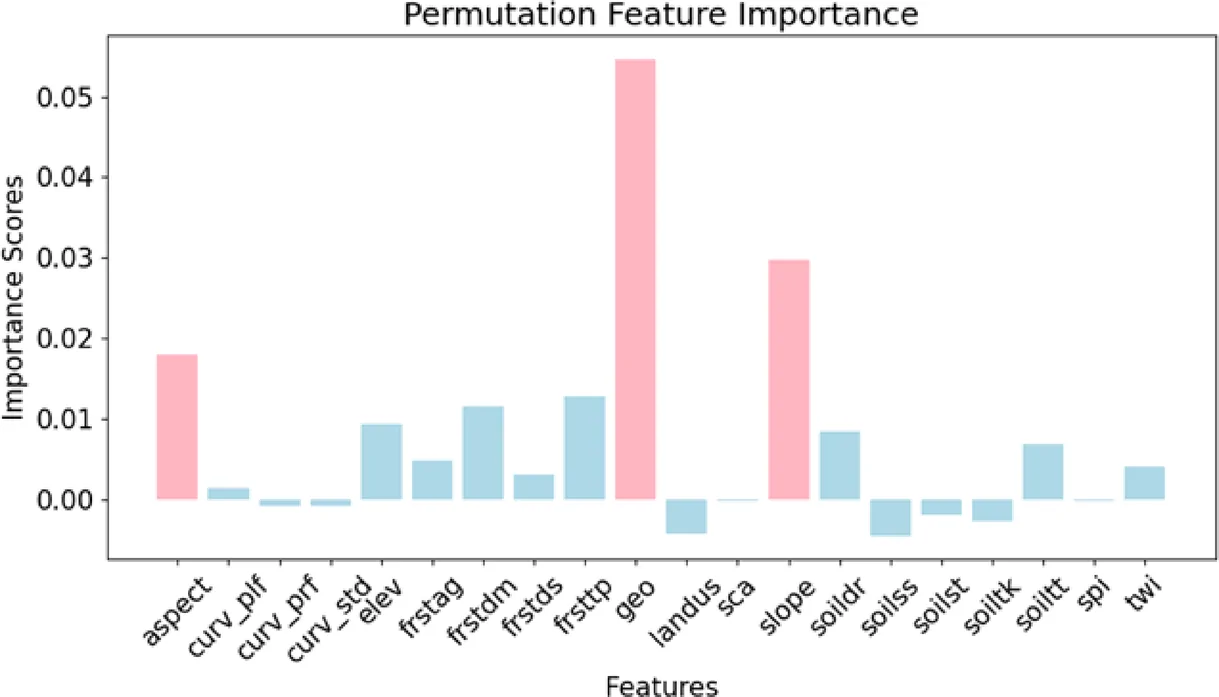
Permutation Feature Importance analysis applied to the CNNs model, identifying and quantifying the relative importance of input conditioning factors. Aspect, forest age, geology, slope, and forest diameter are the most significant factors, represented by red bars.

The training of the CNNs model was performed by extracting patches from the conditioning factors and landslide inventory. However, because the model uses patches as input, feature-importance analysis could not be performed using SHAP; therefore, we additionally applied permutation feature importance^76^. Permutation feature importance involves randomly shuffling the values of one feature at a time, generating predictions with the model, and comparing the resulting accuracy with that obtained when using all factors. This allows us to assess the importance of each feature. In addition, the permutation feature-importance analysis was repeated 100 times, and the average importance scores were calculated. Factors that consistently showed high average importance can therefore be considered reliable contributors to the model’s predictions (Fig. 6).

In the CNNs model, similar to the other machine learning models, geology and slope were identified as the most important factors. Geology showed the highest importance, while the soil series factor showed the lowest or nearly the lowest importance. The third most important factor was aspect.

The results of susceptibility mapping using the trained CNNs showed that, compared to the SVM, RF, and MLP models, a larger portion of the study area was classified as Very Low susceptibility, while only specific areas adjacent to landslide points were classified as High or Very High susceptibility. In addition, the proportion of Moderate susceptibility was lower (Fig. 6(a)). Furthermore, in the test area, which was not included in the training, the proposed model predicted the areas near landslide points as High or Very High susceptibility, whereas the other models underestimated the susceptibility. These results suggest that the CNNs model exhibits less ambiguity in landslide susceptibility prediction compared to the other models. Moreover, the CNNs model successfully identified higher susceptibility around landslide-prone areas in the eastern, southwestern regions and the test area, which were not well captured by the other models. When applied to the test dataset, the model achieved an accuracy of 0.7586, outperforming the other models.

**Fig. 7:**
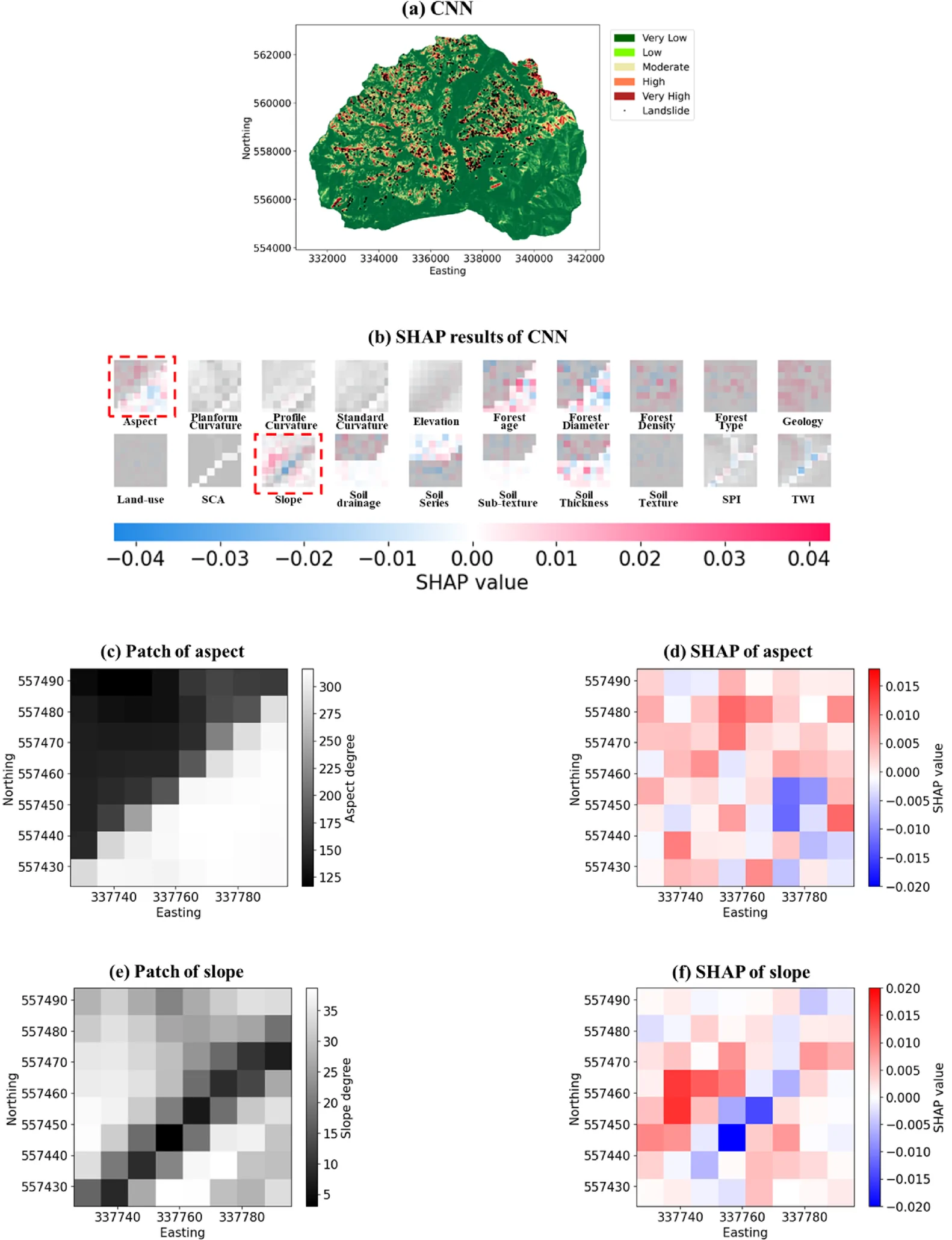
Convolutional Neural Networks (CNNs) results: (**a**) susceptibility mapping, and and (**b**) SHAP visualization, showing spatial importance and contribution of input features to the CNNs prediction output. Red indicates features positively associated with susceptibility, while blue indicates negatively associated regions. (**c**)–(**f**) Detailed SHAP analysis of CNNs input patches. (**c**), (**d**) Aspect factor: SHAP values show reduced or negligible contributions for north-facing slopes (white) and south-facing slopes (black), with increased contributions for east- and west-facing slopes (gray). (**e**), (**f**) Slope factor: SHAP values indicate higher susceptibility contributions for steep slopes (black) compared to gentle slopes (white). The maps were generated using Python 3 with the Matplotlib library (https://matplotlib.org/).

The training of the CNNs model was performed by extracting patches from the conditioning factors and landslide inventory. The patch size was set to 8 × 8, with each cell having dimensions of 10 m along the x-axis and 10 m along the y-axis. Patches were extracted by moving one cell at a time across the entire area, resulting in a total of 644,365 generated patches. The patch size was determined empirically by testing multiple candidate sizes and selecting the one that yielded the most stable performance. When determining the label of a landslide patch, only the central four cells were considered. If any one of the central four cells was a landslide point, the label was set to 1. If none of the central four cells contained a landslide point, the label was set to 0, even if other cells contained landslide points. This patch setting aimed to enable the CNNs model to predict landslide susceptibility more accurately, focusing on the precise location. The appropriate patch size and the size of the central cells to focus on were selected empirically.

The results of susceptibility mapping using the trained CNNs model showed that, compared to the SVM, RF, and MLP models, a larger portion of the study area was classified as Very Low susceptibility, while only specific areas and regions adjacent to landslide points were classified as High or Very High susceptibility. In addition, the proportion of Moderate susceptibility decreased (Fig. 7(a)). These results suggest that the CNNs model exhibits less ambiguity in landslide susceptibility prediction compared to the other models. Moreover, the CNNs model successfully identified higher susceptibility around landslide-prone areas in the eastern, southwestern regions and the test area, which were not well captured by the other models. When applied to the test dataset, the model achieved an accuracy of 0.7586, outperforming the other models.

Subsequently, SHAP analysis was conducted on the trained CNNs model. Applying SHAP to image data allows for the identification of which parts of the input image have a positive influence and which parts have a negative influence. The SHAP results are visualized for each feature individually, with red representing high SHAP values and blue indicating low SHAP values (Fig. 7(b)). According to the permutation feature importance results, aspect and slope were selected for additional analysis, as they can be treated as continuous variables.

Figure 7(c)–(f) represent SHAP value maps specifically separated for the aspect and slope factors, which were identified as important. For the aspect factor, SHAP contributions tended to decrease when the slope faced north, whereas relatively higher SHAP values were observed for west and south-facing slopes (Fig. 7(c), (d)). In the case of the slope factor, a roughly linear relationship was observed, where higher slopes exhibited greater SHAP values and lower slopes showed lesser values (Fig. 7(e), (f)). However, unlike other ML models, SHAP analysis in CNNs must be performed for each individual image patch, which may result in inconsistent patterns depending on the patch. Moreover, the wide dispersion of SHAP values makes interpretation more challenging. Therefore, considering these limitations, a more detailed investigation of these two factors was conducted using Grad-CAM.

**Fig. 8:**
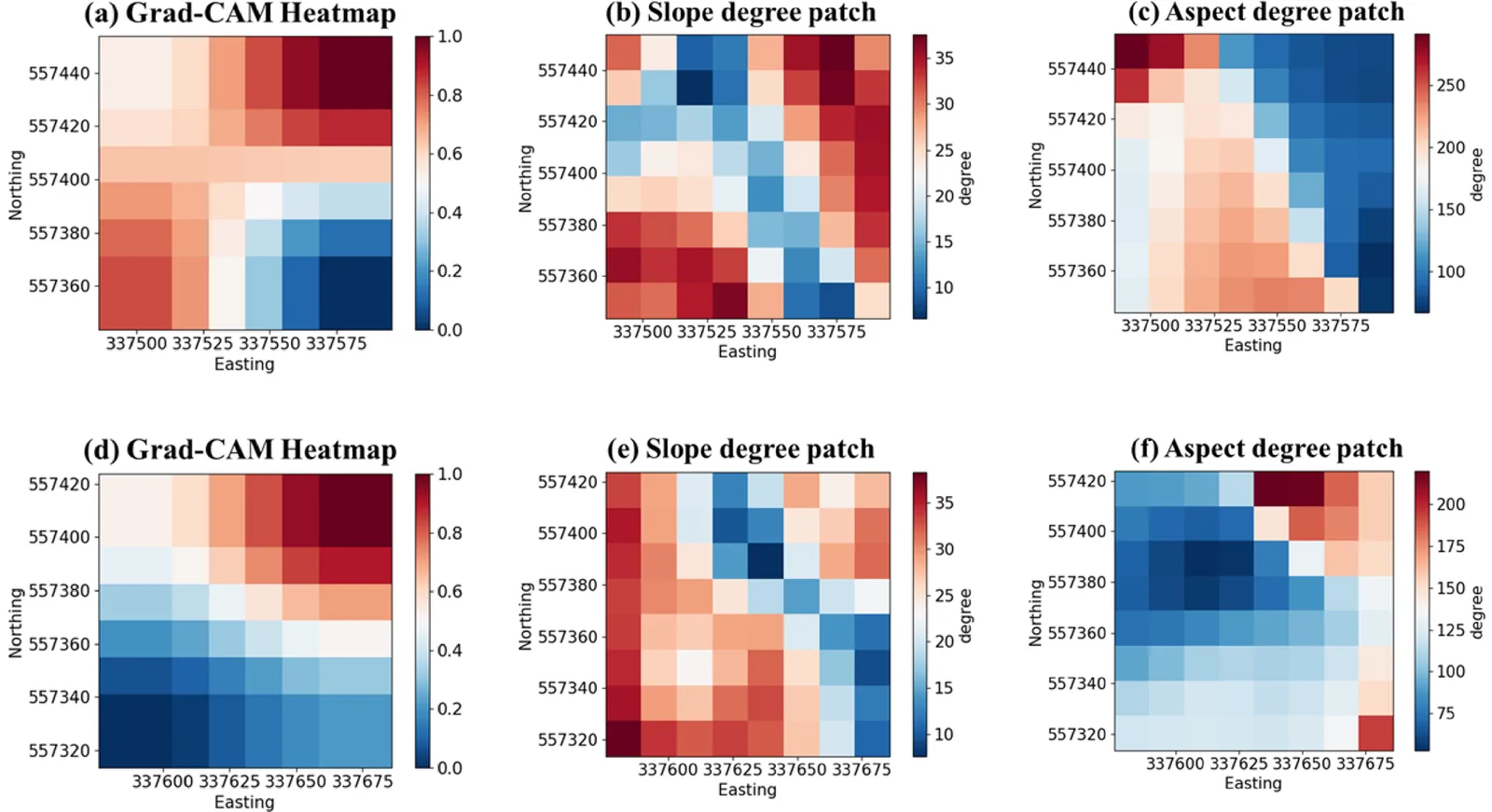
Grad-CAM heatmaps highlighting regions within input patches that significantly influence CNNs predictions for landslide susceptibility. (**a**), (**d**) Grad-CAM heatmap illustrating areas with high contribution to the CNNs output. (**b**), (**c**), and (**e**), (**f**) Corresponding input patches for the aspect and slope factors of (**a**) and (**d**), respectively, demonstrating how the CNNs identifies critical values from conditioning factors. The upper map corresponds to a ridge area, while the lower map represents a valley area.

The Grad-CAM heatmap for the CNNs model can be obtained for each input instance by leveraging all conditioning factors simultaneously, with gradient values computed from one of the model’s layers during output prediction. The specific layer used for generating the heatmap was empirically selected based on which layer provided the most visually interpretable results. Since the heatmap reflects the combined influence of all input factors, potential correlations can be investigated by comparing the spatial patterns of the heatmap with those of individual conditioning factors. In this paper, we analyzed the pattern similarities between the Grad-CAM heatmap and the aspect and slope factors (Fig. 8).

In the upper patch, the slope transitions from steep to gentle as it shifts from the northeastern to southern direction, and the aspect changes from east-facing to southwest-facing, indicating a ridge-like topography (Fig. 8(b), (c)). High heatmap values were observed on the ridge, particularly in areas with steep slopes that face east. Additionally, in the southwestern to northwestern section of the patch, another ridge can be identified through variations in slope and aspect. Although less pronounced than the east-facing ridge, this region also shows a pattern in which heatmap values increase and then decrease (Fig. 8(a)-(c)).

In contrast, the lower patch exhibits patterns of slope and aspect indicative of a valley-like topography (Fig. 8(d)-(f)). In this area, the heatmap values tend to decrease as the aspect shifts from southwest-facing to northeast-facing slopes.

These pattern-based analyses suggest that, within the CNNs model, north-facing slopes are associated with lower landslide susceptibility compared to east, west, or south-facing slopes. Such spatial pattern interpretation, when combined with the SHAP analysis results, contributes to enhancing the interpretability and reliability of the CNNs model’s predictions (Figs. 7 and 8). Furthermore, the high importance attributed to the geology factor in both SHAP and permutation analyses reinforces the credibility of the susceptibility assessment results.

### Performance evaluation

The performance of the models described above was evaluated using four metrics. Among the three ML models, the MLP model demonstrated the highest performance, achieving an accuracy of 0.7034, a Cohen’s kappa score of 0.4069, and an AUC of 0.8024. The SVM and RF models yielded accuracies of 0.6621 and 0.6931, respectively. In contrast, the CNNs model outperformed all other models, with an accuracy of 0.7586, an AUC of 0.8067, a Cohen’s kappa index of 0.5172, and an F1 score of 0.7712, showed competitive results across most key metrics (Table 2). Furthermore, in terms of Recall, which is considered the most appropriate metric for evaluating disaster-related hazards, the CNNs model demonstrated the highest performance at 0.8138. This result indicates that, among all models tested, the CNNs was the most effective at accurately identifying actual landslide events within the test area (Table 2).

**Table 2:** The Performance evaluation of each model.

Accuracy	SVM	RF	MLP	CNNs
	0.6621	0.6931	0.7034	0.7586
AUC	0.7196	0.7778	0.8024	0.8067
Cohen kappa	0.3241	0.3862	0.4069	0.5172
Recall	0.6276	0.6345	0.6828	0.8138
F1 score	0.6500	0.6740	0.6972	0.7712

### Spatial interpretability

To further demonstrate that the trained CNNs model possesses effectively captures spatial context compared to traditional models, an additional analysis was conducted by directly comparing the landslide inventory with the predicted susceptibility values^87,88^. This analysis employed a Distance Decay approach to evaluate how the predicted probability (P_mean_) changes as a function of the distance from the actual landslide locations.

For the 145 landslide points in the test dataset, the vicinity was categorized into four spatial clusters based on a 10 m grid: the landslide cell (Point), 1-ring (10–14 m), 2-ring (15–28 m), and 3-ring (29–42 m). As summarized in Table 3, the CNNs model demonstrated the highest P_mean_ at the actual landslide points (0.6845), showed a higher mean probability than the point-based models—SVM (0.5184), RF (0.5573), and MLP (0.6432). This indicates that the CNNs model identifies the vulnerability of landslide-prone areas with higher confidence, whereas the traditional models exhibit a greater frequency of detection failures at the source point.

Furthermore, considering that landslides are spatial phenomena rather than isolated points, the CNNs model maintained consistently higher P_mean_ values in the surrounding rings compared to other models. Notably, the CNNs exhibited a clear spatial gradient where the predicted values systematically decreased as the distance from the landslide center increased. This systematic reduction confirms that the patch-based learning mechanism of the CNNs effectively captures the localized topographical context and spatial sequences, granting it superior spatial discriminability. Such results align with recent methodological emphasis that a model’s reliability is defined by its ability to concentrate high-risk predictions precisely within and immediately around the observed landslide zones^87,88^.

**Table 3:** The mean P value of each model for distance decay analysis.

Distance	Number of cells	mean *P* value			
		SVM	RF	MLP	CNNs
0 m (landslide)	1	0.5184	0.5573	0.6432	0.6845
1-ring (10–14 m)	8	0.5121	0.5379	0.6491	0.6798
2-ring (15–28 m)	16	0.5065	0.5240	0.6404	0.6627
3-ring (29–42 m)	36	0.4944	0.5118	0.6189	0.6359

## Discussion

The results of this study indicate that specific geological and topographical characteristics significantly influence landslide susceptibility. Based on the geological background, analyses by KIGAM and the Korea Forest Service indicate that the Gi (Gneiss) unit is particularly vulnerable to physical and chemical weathering. This weathering process increases permeability and, during intense rainfall, reduces shear strength, thereby contributing to landslide occurrence. In contrast, while Oj (Ordovician sedimentary rocks) consists of very hard rock, the presence of vertical joints and bedding planes formed by tectonic activity increases the risk of rock-slope failure or rockfall. For TRn2, comprising sandstone, siltstone, and mudstone, the material becomes highly susceptible to moisture; when weakened by water absorption, landslides may occur in the form of plane failure. These findings suggest that although geological units are categorical factors, it is possible to analyze the specific relationship between individual lithologies and landslide susceptibility.

In this study, our model identified a pattern of higher susceptibility on north-facing slopes, a result likely influenced by the climatic and geographical characteristics of Korea. Korea experiences four distinct seasons with specific patterns in wind direction and rainy seasons, which may make certain slope aspects more vulnerable to moisture during specific periods. However, it is important to note that the landslide data used in this paper were derived from a single heavy rainfall event in July 2006. While rainfall is widely recognized as a primary triggering factor, it was not incorporated as an input variable. Instead, our analysis focused on characterizing the influence of pre-existing conditioning factors to assess the fundamental, intrinsic susceptibility of the study area prior to a specific triggering event. Nevertheless, since the data reflect only one extreme event in a single region, applying the model to different time periods or regions may yield different results due to potential overfitting or varying meteorological conditions. Future research should prioritize integrating dynamic rainfall data—such as intensity, duration, and antecedent precipitation—to enhance the temporal accuracy of landslide susceptibility predictions.

Validating landslide susceptibility maps at a regional scale remains challenging due to the lack of ground-truth labels for entire areas. In this study, we addressed this by designating an independent test area for quantitative evaluation and analyzing the spatial discriminability in the vicinity of known landslides. Our findings confirmed that the CNNs model outperforms traditional methods in capturing local spatial features. Furthermore, the reliability of the proposed framework was reinforced through Explainable AI (XAI) techniques. By simultaneously applying SHAP for global feature importance and Grad-CAM for spatial focus analysis, we verified that dominant factors such as slope and aspect are closely aligned with the actual environmental drivers of landslides in the study area. This dual suggests that the model is capable of both high predictive accuracy and geological consistency, moving beyond a “black-box” approach to a more transparent and scientifically grounded landslide assessment.

Although CNNs demonstrated superior predictive performance, we acknowledge that this result could be influenced by disparities in training data quantities (CNNs: 6,348 samples; RF, SVM, and MLP: 1,780 samples). Further experiments that control for sample size are required to strictly attribute observed differences to model architecture rather than data availability.

Furthermore, a specific adjustment was made to the evaluation process to ensure a fair comparison. While the CNNs was trained using the central 2 × 2 pixels (4 cells) of each patch as labels to capture spatial context, for testing, we restricted the label to only the single centermost pixel (at position 5,5) to match the point-based test set used for the RF, SVM, and MLP models (*n* = 290). Interestingly, when we evaluated the CNNs using the original 2 × 2 block labeling for the test set, the model exhibited even higher predictive accuracy. However, we maintained the single-pixel labeling approach for the final results to ensure that all models were evaluated on an identical number of test samples, thereby providing a more conservative and consistent comparative framework.

Despite the encouraging findings, we acknowledge several limitations that warrant further investigation. Our model was trained and tested solely on data from a single geographic region, so its applicability to other environmental or geological contexts remains unverified. Consequently, direct cross‑regional deployment may be constrained. Future research should evaluate the model’s robustness across diverse regions and explore transfer‑learning approaches to mitigate these spatial limitations.

## Conclusion

In this paper, we sought to improve the accuracy of landslide susceptibility predictions using CNNs models, which are recently evaluated to outperform ML models. In summary, the proposed CNNs demonstrated superior predictive performance across both accuracy and recall compared with the SVM, RF, and MLP baselines. Specifically, accuracies were 0.7586 for CNNs, versus 0.6621 (SVM), 0.6931 (RF), and 0.7034 (MLP). For hazard-related recall, the CNNs achieved 0.8138, outperforming SVM (0.6276), RF (0.6345), and MLP (0.6828). These results support the main conclusion that the CNNs’ patch-based spatial learning yields higher hazard prediction performance and better spatial discriminability, enabling potentially enhancing the identification of landslide-prone areas and contributing to the reduction of missed events in disaster management applications. However, despite their high performance, CNNs models are considered black box models, raising concerns about the reliability of their results. To address these concerns, we applied eXplainable Artificial Intelligence (XAI) algorithms, such as SHAP and Grad-CAM, to the CNNs model.

The SHAP analysis of the ML models revealed that geology had the greatest influence on model predictions, followed by slope and soil series. For the geology factor, a significant number of landslide occurrences were concentrated within specific geological classes, and those classes with the highest frequency of landslides were assigned correspondingly high SHAP values. The slope factor exhibited a roughly linear relationship between the magnitude of slope values and their corresponding SHAP values, indicating that higher slopes contributed more strongly to landslide susceptibility predictions. In the case of the soil series factor, 54 different soil types are distributed within the study area, and there are 405 types in South Korea overall. Given this diversity, it was determined that an analysis of the 54 types would not be generally applicable. Therefore, further analysis was conducted on the aspect factor, which showed relatively high SHAP values and consistent patterns across models, compared to other factors. the aspect factor showed a consistent pattern: north-facing slopes were associated with lower SHAP values, while east and west-facing slopes exhibited moderate SHAP values, and south-facing slopes showed the highest contributions.

Since SHAP does not inherently provide a ranked order of feature importance for CNNs, permutation feature importance was additionally applied. The results confirmed that the three most important factors were geology, slope, and aspect. The relationships between the slope and aspect values and their corresponding SHAP values were also examined. Consistent with the previous SHAP results, aspect had a greater influence when facing south, east, or west than when facing north, and steeper slopes were associated with higher susceptibility values.

Further spatial analysis was conducted by applying Grad-CAM to image patches located in ridge and valley areas. In ridge-type topographies, higher heatmap values were observed in areas with steep slopes and east, south, or west-facing aspects, while lower values were found in north-facing or more gently sloped areas. In valley-type patches, a similar trend was observed: heatmap values decreased as the slope became gentler, accompanied by a gradual shift in aspect direction from southwest-facing to northeast-facing. These spatial patterns indicate that in the study area, landslide susceptibility is strongly influenced by a combination of geology, slope, and aspect, supporting the interpretability of the CNNs outputs. Additionally, when evaluated using four quantitative performance metrics, the CNNs demonstrated consistently higher performance than the other models across the major evaluation metrics. In predicting susceptibility maps, the CNNs identified landslides in the test area more successfully than the other models. These results suggest that utilizing spatial patterns have the potential to improve the generalization capability of DL models.

Ultimately, these results not only demonstrate the high performance of the CNNs model but also enhance the reliability of the predictions by conducting an in-depth interpretability analysis using explainable AI (XAI) techniques for both ML and DL models. However, since the current study was conducted using spatial partitions within a single region, further validation is required by applying the proposed approach to other geographic areas in future research.

## Data Availability

The data shown in this article are available from the corresponding authors upon a reasonable request.
